# The Role of Physical Exercise in Inflammatory Bowel Disease

**DOI:** 10.1155/2014/429031

**Published:** 2014-04-30

**Authors:** Jan Bilski, Bartosz Brzozowski, Agnieszka Mazur-Bialy, Zbigniew Sliwowski, Tomasz Brzozowski

**Affiliations:** ^1^Department of Ergonomics and Exercise Physiology, Faculty of Health Sciences, Jagiellonian University Medical College, 31-531 Cracow, Poland; ^2^Gastroenterology Clinic, Jagiellonian University Medical College, 31-501 Cracow, Poland; ^3^Department of Physiology, Faculty of Medicine Jagiellonian University Medical College, 31-531 Cracow, Poland

## Abstract

We reviewed and analyzed the relationship between physical exercise and inflammatory bowel disease (IBD) which covers a group of chronic, relapsing, and remitting intestinal disorders including Crohn's disease (CD) and ulcerative colitis. The etiology of IBD likely involves a combination of genetic predisposition and environmental risk factors. Physical training has been suggested to be protective against the onset of IBD, but there are inconsistencies in the findings of the published literature. Hypertrophy of the mesenteric white adipose tissue (mWAT) is recognized as a characteristic feature of CD, but its importance for the perpetuation of onset of this intestinal disease is unknown. Adipocytes synthesize proinflammatory and anti-inflammatory cytokines. Hypertrophy of mWAT could play a role as a barrier to the inflammatory process, but recent data suggest that deregulation of adipokine secretion is involved in the pathogenesis of CD. Adipocytokines and macrophage mediators perpetuate the intestinal inflammatory process, leading to mucosal ulcerations along the mesenteric border, a typical feature of CD. Contracting skeletal muscles release biologically active myokines, known to exert the direct anti-inflammatory effects, and inhibit the release of proinflammatory mediators from visceral fat. Further research is required to confirm these observations and establish exercise regimes for IBD patients.

## 1. Introduction

Chronic inflammation plays a central role in the pathology of many diseases. For some, such as rheumatoid arthritis (RA), inflammatory bowel diseases (IBD), and asthma, the most characteristic is a massive infiltration of inflammatory cells at the site of disease activity and the local presence of inflammatory mediators in elevated concentrations as well as their abundance in the systemic circulation. These may lead to pathological disorders in organs distant from the primary inflammatory lesions probably evoked by systemic inflammatory mediators [1].

Ulcerative colitis (UC) and Crohn's disease (CD) are the two main forms of IBD characterized by a cyclic nature, alternating between active and quiescent states impairing patients' quality of life. Although the inflammatory process in CD is typically transmural and can affect any part of the gastrointestinal tract, the UC affects only the colon and is limited to the mucosa and superficial submucosa [2]. Anorexia, malnutrition, altered body composition, and development of mesenteric white adipose tissue (mWAT) hypertrophy (accumulation of intra-abdominal mWAT) are other well-known features of IBD and especially of CD [2]. Additionally, apart from intestinal changes in CD, the secondary disorders in distant organs such as skin lesion, arthritis, osteoporosis, eye, and liver disorders may frequently occur [2]. Association between inflammation and carcinogenesis is well proven and IBD is an important risk factor for the development of colon cancer [3]. Although progress has been made in understanding the IBD, its etiology is still unknown. The accepted theory suggests that a combination of environmental agents and a dysfunctional mucosal immune system in genetically susceptible individuals could lead to the development of either CD or UC [4, 5]. It was established that CD is a Th1 cytokine-mediated disease characterized by increased production of interferon (IFN-) *γ*, whereas UC most likely resembles a modified Th2 profile, characterized by increased production of interleukin- (IL-) 5 production and normal IFN-*γ* production [6]. Cytokines, such as TNF-*α*, IL-1*β*, and IL-6 are more promiscuous in their function because they are associated with both forms of IBD to a lesser or greater degree [6, 7]. Each of these cytokines can activate NF-*κ*B and the mitogen-activated protein kinases, thereby initiating a cascade of “downstream” proinflammatory effects that are the immediate prerequisites of tissue and organ pathology in IBD [6]. Recently, TNF-like weak inducer of apoptosis (TWEAK), the TNF super family (TNFSF) member, has appeared as a new factor in the inflammatory processes and its crucial role in IBD has been proposed [8–10]. Its actions are mediated by binding to fibroblast growth factor-inducible 14 (Fn14), surface receptor that has been linked to several intracellular signaling pathways, including the nuclear factor-*κ*B (NF-*κ*B) inflammatory pathway [11]. Fn14 can be upregulated on intestinal epithelial cells, most probably by their exposure to microbial or inflammatory products and contribute to failure of the mucosal barrier; the induction of IEC-derived mediators that promote chronic inflammation and shape gut immunity against commensally bacteria [9]. Interestingly, this expression of Fn14 is upregulated in CD patients [11].

The concept of exercise training could cover heterogeneous interventions that differ in type (e.g., endurance versus resistance training, acute versus chronic exercise), intensity, frequency, and duration. Structured exercise training could consist of aerobic exercise, resistance training, or a combination of both. In contrast to structured exercise training, physical activity is defined as any bodily movement produced by skeletal muscle contractions resulting in increased energy expenditure [12].

The well-documented observations that physical activity is inversely correlated with systemic low-level inflammation lead to suggestion that the anti-inflammatory activity induced by regular exercise may be responsible for some beneficial health effects in patients with chronic diseases [13]. However, the intensive exercise may induce a transient mild systemic inflammation and increased level of cytokines and these adverse effects depend on intensity and duration of exercise. Following prolonged and strenuous exercise, the function of the immune system is impaired for several hours [14, 15], but on the other hand, the regular exercise training has been shown to increase resistance to infections [16–19].

The possible beneficial effects of physical activity on the gastrointestinal tract have been so far little studied. It is known that intensive exercise such as long distance running and triathlons could cause nausea, heartburn, diarrhea, or even gastrointestinal bleeding and marathon runners suffer from “runner's ischemic colitis,” involving bloody diarrhea, fatigue, and fever [20]. The involvement of physical activity in prevention of colon cancer [21] has been well proven, but the effect of physical activity on IBD is less documented and the importance of exercise as adjunct anti-inflammatory therapy has been suggested [22–24]. There is a general commitment that IBD patients could benefit from physical training because regular exercise could improve psychological health and reduce some disease symptoms and complications [25]. However, from our current knowledge on exercise in relation to immune functions, cytokines network, and in particular, the secretory role of contracting muscles, one concludes that a relatively small attention has been addressed to mechanisms of action of regular exercise in patients with a chronic inflammatory disease like IBD, in which immune response is impaired. Most studies have examined the beneficial effects of exercise in terms of quality of life and general fitness but relatively few studied effects of training on disease pathogenesis and measures of inflammation and evidence-based recommendations could not yet be made for this disease [22–25].

Taking into the account the lack of sufficient evidence-based exercise guidelines for those subjects who suffer from IBD and that physical activity is recommended for such patients as a complementary medical therapy, the purpose of the review was to evaluate published human and experimental research studies focusing on physical activity and IBD.

Particular aims of the present review were to update the literature on physical activity in IBD and to evaluate (1) whether physical activity in the preillness period could influence onset of IBD, (2) impact of exercise on IBD, and (3) overview on selection of types and intensities of exercise in individuals suffering from this disease and how exercise affects experimental models of this disease. In discussion, we focused on potential mechanisms of beneficial action of physical training.

## 2. Methods

### 2.1. Search Strategy

We searched the following electronic databases up to January 2014: MEDLINE (accessed by PubMed), Google Scholar, Web of Science, PEDRo, and IBD/FBD Group Specialized Register (June 2013). The following search string was entered into each database using MeSH terms, appropriate alternatives, and Boolean operators: inflammatory bowel disease, Crohn's disease, or ulcerative colitis and exercise, physical activity, physical fitness, or physical training. Variations of the above search strategy were used to search the databases other than Medline.

### 2.2. Study Selection

**Figure 1:**
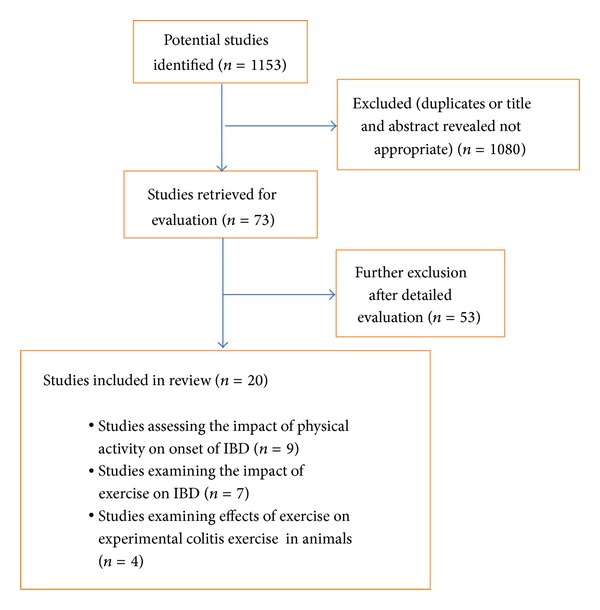
Flowchart for selection of studies.

**Figure 2:**
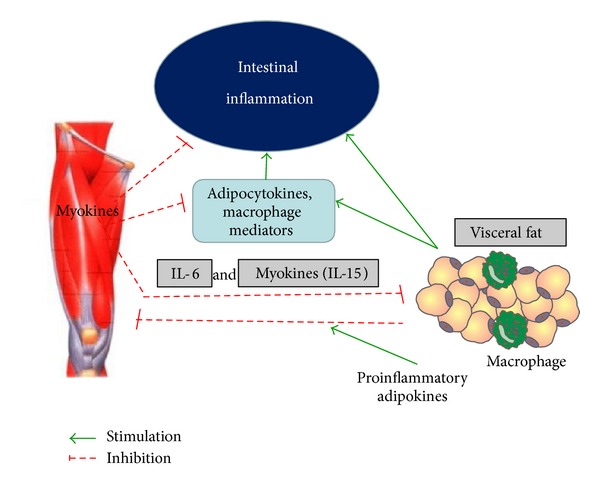
Crosstalk between skeletal muscle, adipose tissue, and intestinal inflammation.

Two reviewers (JB and TB) independently assessed study eligibility and the full-text articles were then examined. Bibliographies from existing articles were screened manually. Only fully published papers were reviewed.  Figure 1 provides a flowchart of study selection. In Figure 2, the insight into mechanism of crosstalk between skeletal muscle, adipose tissue and the inflammation in the gut is presented.

## 3. Results

### 3.1. Exercise in the Preillness Period

The incidence and prevalence of IBD rapidly increased in last years in developed countries and the rise witnessed in the rest of the world closely correlates with adopting a western lifestyle [26]. These observations support the notion that a variety of environmental factors contribute to the pathogenesis of intestinal diseases [26]. In developed countries, peoples' lifestyle has changed significantly, being affected by serious modifications in dietary habits and physical inactivity [27]. Those changes in lifestyle may have a bearing on the course of the disease. Some studies have examined the effect of lifestyle and particularly physical activity as supposed by causal agents for the onset of IBD (Table 1).

The protective role of physical activity against the onset of IBD was first postulated by Sonnenberg [28] who found that occupations characterized by outdoor physical work appeared to be protective compared with those occupations classified as sedentary in retrospective study in male and female German workers. In small (*n* = 725) case-control study Persson et al. [29] reported an inverse association between physical activity and CD (but not UC) onset in a Swedish cohort. A prospective study which followed two cohorts, each of more than 2.3 million persons in Denmark, for 5 or 10 years, found a small association between sedentary office work lifestyle and the onset of IBD [30]. In 1998, a study by Klein et al. [31] determined that IBD patients (*n* = 88) had lower levels of physical activity during their preillness period than clinic controls (*n* = 68; *P* < 0.001). IBD patients had lower levels of physical activity during their preillness period compared to clinic but not population controls. Cucino and Sonnenberg [32] examined the occupations of IBD mortalities between 1991 and 1996 in 2399 CD and 2419 UC patients in USA and found that IBD mortality was low in occupations associated with manual work and relatively high in sedentary occupations. When Halfvarson et al. [33] studied environmental factors in a population-based Swedish-Danish twin cohort using the cotwin control method, they found no significant differences between the twins in physical activity before the diagnosis of IBD.

In a recent the European Prospective Investigation into Cancer and Nutrition study, 300,724 participants were recruited into and the cohort was monitored identifying participants who developed CD or UC, but physical activity did not show any association with UC or CD [34]. In case-control study in Slovakia [35] which included 338 patients (190 CD, 148 UC) and 355 controls CD and UC, an onset of IBD was inversely associated with physical activity. In another recent study in two large prospective cohorts of US women (194 711 women) the association between physical activity and risk of ulcerative colitis and Crohn's disease was studied [36]. Women enrolled in this prospective cohort study provided data on physical activity since 1984 in Nurses' Health Study and 1989 in Nurses' Health Study II and followed up through 2010 (USA). Physical activity was found inversely associated with risk of Crohn's disease but not of ulcerative colitis.

The physical activity in preillness period was shown to reduce risk of the onset of IBD and this reduction was found to be stronger for CD than UC [29, 36].

### 3.2. Impact of Exercise on IBD

The physical activity has been used in IBD as an adjunctive therapy regime, although the effectiveness of exercise on disease activity has not been well described and the mechanisms of its potential beneficial effects are poorly understood [25, 37]. Studies that examined the effect of exercise on IBD involved mainly patients with quiescent state of disease (Table 2). In sedentary patients with inactive or mildly active CD, the moderate exercise by means of walking program or yoga led to significant improvement in measures of quality of life and stress levels [38–41]. The moderate-intensity exercise was well tolerated by IBD patients who are in remission and did not provoke subjective symptoms or changes in gastrointestinal parameters [42, 43]. Ploeger et al. [44] tested the effect of moderate intensity continuous exercise and high intensity intermittent exercise in youth with CD and concluded that such patients can engage in different types of exercise without a significant exacerbation of the disease. The results of the UK online survey have shown that a majority of respondents were undertaking regular exercise which was found to be beneficial for the symptoms of IBD. However, most of the respondents were prone to stop exercising at some point because of their increased incidents of complaints on severity of symptoms [45].

### 3.3. Intestinal Disorders and Physical Activity in Experimental Animals

Some of these aspects were also observed at experimental conditions because in mouse model of colitis, the forced treadmill exercise training exacerbated inflammation and increased mortality, while voluntary wheel training was protective in this rodent model [46]. This effect of treadmill exercise was accompanied by increased morbidity due to excessive diarrhea episodes and mortality [46]. In contrast, thirty days of voluntary wheel running attenuated inflammatory gene expression in the distal colon reduced the diarrhea incidences and protected mice from colitis-induced morbidity [42]. Moreover, the induction of experimental colitis by TNBS or dextran sulfide administration caused a significant increase in the TNF-content in the colonic mucosa and submucosa [47, 48]. In another study, the long-term physical exercise of 6-week running attenuated the colonic TNF-*α* protein content indicating an anti-inflammatory impact of exercise training [49]. Some studies have shown that both forced treadmill and voluntary wheel exercise training can exert an anti-inflammatory effect in the inflamed colon [48–51]. In study by Saxena et al. [48] exercise training (treadmill running at gradually increasing speeds 10, 12, 16, and 18 m/min and a 5% incline for 20 min) significantly decreased proinflammatory cytokines in the adiponectin knockout mice with dextran sodium sulfate induced experimental colitis. In another study moderate exercise (30 min per day of swimming) attenuated chronic stress-induced intestinal barrier dysfunction in mice, possibly due to augmentation of antimicrobial responses in the small intestine [52]. A summary of experimental studies on the experimental IBD and exercise depending on its intensity is shown in Table 3.

## 4. Discussion

These findings point to the important role for exercise in the adjunct treatment of IBD in humans. Since IBD affects up to 0.25% of the US population or ~750.000 people; thus it is considered as a significant problem [53]. The fact that selection of a proper dosage of the exercise was able to alleviate colitis symptoms, reduce colon inflammation, and counteract the adverse effects associated with pharmacological therapy (e.g., 5-aminosalicylic acid) [47–49] seems to be of key interest in convincing medical professionals to adopt the life style strategy as an adjunct therapy in their IBD treatment.

The protective effect of exercise may to some extent be attributed to its anti-inflammatory effect and it may mediate via muscle-derived peptides, the so-called “myokines” [54]. Contracting skeletal muscles release myokines like IL-15 with endocrine effects, mediating direct anti-inflammatory effects, and/or specific effects on visceral fat [54]. Possible role of a new myokine irisin which is released during exercise and act on the white fat cells is still studied [54].

Creeping fat in patients with Crohn's disease refers to hypertrophy of the mesenteric fat tissue located around the inflamed parts of the intestine [55] and recent research suggests that this fat wrapping contribute actively to disease severity and may influence onset of complications [56–63]. Accumulating evidence suggests that mesenteric white adipose tissue (mWAT), composed of not only fat but also macrophages and T lymphocytes, plays an important role as the source of inflammation and releases various inflammatory factors such as cytokines and chemokines [64–68]. This mesenteric fat which is present from the onset of disease is associated with overexpression of TNF-*α*, leptin, and other adipokines and correlates with the severity of intestinal inflammation and tissue injury, suggesting an important role for adipose tissue in the intestinal inflammatory process in CD [55, 62, 69–71]. The intestinal luminal leptin, a cytokine produced by adipocytes, is increased in CD and can upregulate NF-*κ*B expression in colonic epithelial cells [58, 72–74]. Leptin is considered to be a proinflammatory cytokine [58] and directly regulates production of several cytokines, particularly those released from T cells. It increases IL-2 and interferon *γ* production while decreasing IL-4 levels [58]. An overexpression of leptin mRNA in mWAT was reported in IBD patients, indicating that leptin might participate in the inflammatory process by enhancing mesenteric TNF-*α* expression [75, 76] and leptin levels have been shown to be significantly higher in mesenteric adipose tissue from CD patients, than in patients with noninflammatory disease [58, 72]. Experimental colitis in rats resulted in elevated circulating leptin levels which correlate with the degree of inflammation and the development of anorexia [77] and leptin antagonist ameliorated the development of chronic experimental colitis [78]. Another adipokine, adiponectin, which is considered anti-inflammatory, has a structure similar to TNF-*α* but antagonizes its effects by reducing secretion and attenuating the biological actions by competing for the receptor [79–81]. Divergent data have been presented about circulating levels of adiponectin in patients with IBD [72, 74, 80, 82–86]. Adiponectin production is enhanced in hypertrophied mWAT which remained in contact with the intestine of CD patients, and this increase may be specifically related to inflammation and the presence of this fat wrapping [79, 87]. A role for hypertrophied mesenteric fat tissue as a barrier to the inflammatory process was postulated in other studies [86, 88, 89]. However, recent observations of lower levels of serum and mesenteric adiponectin in active CD patients compared with those in remission suggest a defective regulation of anti-inflammatory pathways in CD pathogenesis [80]. The impaired balance between proinflammatory and anti-inflammatory factors due to an increase in secretion of TNF-*α*, leptin, and the release of chemoattractant protein-1 (MCP-1) and the decreased production of adiponectin could result in macrophage accumulation in adipocytes and an inflammatory transformation of the visceral adipose tissue, leading to the appearance of creeping fat [88]. Adipocytokines and macrophage mediators secreted by the creeping fat could further increase the intestinal inflammatory process, leading to mucosal ulcerations along the mesenteric border, a typical feature of CD [82, 90]. The massive cytokine production in the inflamed colon, in addition to translocalizing bacteria, could further induce the production of proinflammatory mediators in the adjacent adipose tissue, thus inducing a vicious cycle, in which inflammatory conditions in the intestine and the mesenteric fat support each other [88, 91]. Cytokine overproduction and particularly leptin by mesenteric fat could lead to anorexia, another feature present in CD [86].

### 4.1. Importance of Skeletal Muscle Crosstalk with the Fat Tissue and the Gut in Protection against Intestinal Disorders

Exercise could improve nutrient metabolism in skeletal muscle as well as vascular function and microcirculation, but the accumulated so far evidence suggests that the protective effect of exercise may to some extent be ascribed to its anti-inflammatory effect. Exercise may exert its anti-inflammatory effect via a reduction in visceral fat mass and/or by induction of an anti-inflammatory environment with each bout of exercise. Such effects may in part be mediated via muscle-derived peptides [54]. If the endocrine and paracrine functions of the muscle are not stimulated through contractions, this will cause dysfunction of several organs and tissues of the body as well as an increased risk of chronic inflammatory diseases [54, 92, 93]. As mentioned before, myokines may balance and counteract the effects of adipokines taking part in crosstalk between skeletal muscle and adipose tissue [37, 54]. The secreted myokines are associated with muscle function revealing a novel secretory proteins released from skeletal muscles during exercise that also have been shown to be impaired with ageing.

The prototype myokine, IL-6, appears to be able to mediate metabolic effects as well as anti-inflammatory effects. In response to muscle contractions muscle fibres express the myokine IL-6, which exerts its effects both locally within the muscle and in several distant organs [54, 94]. It has been accepted that the rise in IL-6 level was a consequence of immune response to local damage observed during exercise. Nowadays it is known that muscle is unique in its ability to produce IL-6 during contraction in completely TGF-independent mode. This suggests a major role for this cytokine in a regulation of metabolism rather than acting as an inflammatory mediator [95, 96]. It was shown that IL-6 released by muscle during exercise can mediate release of GLP-1 from intestinal L cells (and from pancreatic A cells) which in turn acts as an incretin causing insulin release providing an evidence that there is possible crosstalk between adipocytes, muscle, and pancreas responsible for energy homeostasis [96]. The exercise increased dramatically level of IL-6 in mice and induced a parallel marked rise in GLP-1 [96]. Glucagon-like peptides are trophic growth factors that enhance repair of damaged intestinal mucosa and the release of these factors could be in part responsible for beneficial effect of exercise [97–100].

IL-15 that is expressed in human skeletal muscle has been identified as an anabolic factor in muscle growth and has been implicated in muscle-fat crosstalk [54]. It was demonstrated that IL-15 mRNA levels were upregulated in human skeletal muscle following a bout of strength training, suggesting that IL-15 may accumulate within the muscle as a consequence of regular training [101]. Interestingly, a decrease in visceral fat mass, but not subcutaneous fat mass, was observed, when IL-15 was overexpressed in murine muscle. Also the elevated circulating levels of IL-15 resulted in significant reductions in body fat and increased bone mineral content [102, 103]. In a recent study, Boström and colleagues [104] identified a new myokine which they called irisin. This myokine is released during exercise and cause the transformation of white fat cells into bright cells (brown-in-white fat cells), with a phenotype similar to that of brown fat cells [104, 105]. In humans, plasma levels of irisin after 10 weeks of regular endurance training were significantly and markedly increased. It was suggested that irisin could be therapeutic for human metabolic disease, obesity, and other disorders in which the exercise is beneficial [104, 105]. Recently, a new myokine secreted protein acidic and rich in cysteine (SPARC) was functionally characterized [93]. SPARC had increased in skeletal muscle and had been secreted into the circulation in response to exercise. The release of SPARC was linked with inhibition of colon tumorigenesis by increasing apoptosis [106]. SPARC is a secreted matricellular glycoprotein that is involved in the development, remodeling, and tissue repair by modulating cell-cell and cell-matrix interactions as well as other functions such as antitumorigenesis action [106]. Interestingly, a single bout of exercise rapidly increased SPARC blood plasma and muscle levels suggesting that the muscle cells secrete this myokine into the circulation. This exercise-induced increase in SPARC appeared to be muscle-specific because no increase was observed in other organs where SPARC was found to be abundant [106].

Depletion of skeletal muscle mass, decreased muscle strength and endurance, and reduced height velocity in children are characteristic features in IBD [2, 107–109]. Particularly in CD, muscle mass and function are reduced compared to healthy controls, potentially resulting in disability [110]. Mechanisms contributing to muscle impairment and thus potential therapeutic targets are poorly understood. The IBD-related growth failure and decreased muscle mass could be the result of a variety of mechanisms including decreased nutrient intake, malabsorption of ingested nutrients, and increased metabolic rate but also could be attributed to elevated concentrations of inflammatory cytokines, decreased level of insulin-like growth factor 1 (IGF-1), and treatment with corticosteroids [111]. Both plasma IGF-I and muscle IGF-I are decreased in response to diverse inflammatory insults that accelerate the loss of muscle protein [112]. The function of the GH-insulin-like growth factor- (IGF-) I axis depends on finely tuned mechanisms, which can be impaired by inflammatory cytokines released from pathologically modified mWAT. Inflammatory cytokines, notably TNF-*α*, reduce liver GH receptor numbers and seem to be responsible for hepatic GH resistance and decrease of circulating IGF-I level that leads to growth inhibition and decrease of lean body mass (LBM) [112–114]. Recent study has shown an attenuated muscle hypertrophy pathway in CD compared with controls particularly in human subjects with lower serum vitamin D_3_ and lower physical activity indices. This reduced muscle mass in CD may be explained, in part, by impaired activation of muscle protein synthesis pathways, in particular IGF1-Akt pathway. Finally, it was concluded that the normal vitamin D levels and regular exercise may be protective in CD [110]. Studies performed on rats with experimental colitis have demonstrated inhibitory effects of inflammation on IGF-I generation and the linear growth, the mechanisms independent of malnutrition [115, 116]. Impaired function of satellite cells is another link between impaired insulin/IGF-I signaling and muscle protein loss [115]. It was shown that resistance training can prevent and even reverse the progression of sarcopenia [117].

Accumulating evidence suggests that peroxisome proliferator-activated receptor *γ* coactivator *α* (PGC-1*α*) and TWEAK-Fn14 system are key regulators of skeletal muscle mass in various catabolic states. While the activation of TWEAK-Fn14 signaling causes muscle wasting, PGC-1*α* preserves skeletal muscle mass. Inflammatory reactions during IBD favor TWEAK-Fn14 system when physical exercise possibly exhibits a counteractive effect [9, 10, 118, 119].

### 4.2. Other Benefits of Exercise for IBD Patients

Beside the anti-inflammatory actions, several other benefits of physical exercise in IBD patients have been suggested. Ankylosing spondylitis has been specifically associated with IBD, but the exercise therapy improved the flexibility strength and reduced pain of the joints [120]. IBD is associated with decreased bone mineral density and increased risk of osteoporosis [121] and preventive role of exercise has been proposed [122–124]. It was demonstrated that physical exercise can increase bone mineral density (BMD) in CD and may reduce the risk of osteoporotic fracture [122]. The pediatric patients, particularly with Crohn's disease, are at risk for extra intestinal manifestation including growth failure, weight loss, and anemia. Additional beneficial effect of exercise could be amelioration of accompanying anorexia, possibly by modification of the release of adipokines and ghrelin [125]. The reduced food intake in IBD could be caused by abdominal pain, diarrhea and incontinence, surgery, nausea, depression, or a feeling of general unwellness but satiety control in IBD patients is also probably modulated by availability of inflammatory cytokines like leptin which suppresses appetite, reducing the motivation to eat [126–128]. The fatigue is a commonly observed symptom in CD, even in quiescent state of the disease and this effect is probably mediated, at least in part, by cytokines [93, 129]. Role of exercise in reduction of fatigue in chronic diseases including IBD was emphasized [93, 129, 130]. IBD patients have higher levels of daily stress and a lower quality of life compared with general population but also with those patients who suffer from other chronic diseases [27, 28]. The beneficial role of exercise in such cases has been proven [131, 132].

The effect of exercise on different immune parameters could depend on its intensity, duration, and the type of exercise (e.g., endurance versus resistance training, acute versus chronic exercise). Systematic exercise may be beneficial for CD patients for its anti-inflammatory and anabolic properties. However, acute, strenuous exercise could lead to release of inflammatory cytokines that could be involved in the pathology of CD and even induce an exacerbated inflammatory response [24].

Previous studies show that low-intensity exercise is well tolerated in IBD patients. Moreover, this exerted a beneficial effect on course of this disease. In guidelines created in 1998 specifically for IBD patients, physical exercise was recommended for general health to counteract muscle wasting and improve bone density [133]. An aerobic activity for 20 min to 60 min two to five days every week, accompanied by resistance exercise at least twice per week was recommended. The guidelines, however, were not based on actual research. In recent review authors proposed similar recommendations and suggested that two main types of physical interventions should be recommended for CD patients, namely, the aerobic activity and the muscular resistance training [23].

### 4.3. High-Intensity Physical Training in the Treatment of IBD

Single bouts of exercise could activate the same inflammatory mediators as those involved in pathology of IBD. It is generally accepted that high intensity exercise may lead to an acute although transient, exacerbation of inflammation, and the symptoms of IBD. Therefore, such training is generally not recommended for IBD patients [24]. However, such recommendations are not well supported by research studies. Only one study which examined the effect of high intensity intermittent exercise in pediatric patients concluded that that such intense exercise is well tolerated [44].

The generally accepted model for exercise prescription in many chronic inflammatory diseases was moderate-intensity-aerobic continuous training with such proven benefits like increase in exercise capacity, the amelioration of stress, and an increase in quality of life. Recently, however, a body of evidence has indicated that high intensity interval training can be performed safely and lead to similar health effects compared to longer, continuous exercise and is less associated with release of inflammatory mediators [134, 135]. Such exercise program may be particularly beneficial for children with CD, not only to improve exercise capacity, but also because of anabolic action and stimulation of growth and development [136, 137]. On the other hand moderate intensity exercise could be more effective than high intensity in stimulation of release of myokines as shown by Yeo et al. [138].

## 5. Conclusion

Although anti-inflammatory pharmaceutical treatments are beneficial in reducing IBD symptoms, they are often related to serious side effects and their efficacy is not complete. IBD patients continue to have physical and psychological complaints, impairing their quality of life. Preliminary studies demonstrate that moderate exercise has no negative health effects and may diminish some symptoms of IBD. The exercise is recommended also because it could counteract some IBD specific complications by improving immunological response, psychological health, nutritional status, bone mineral density and reversing the decrease of muscle mass and strength. Recent research suggests that the beneficial effects of regular exercise may be in part due to the anti-inflammatory effects of myokines released due to skeletal muscle contractions. Further studies are definitely required to confirm these observations and establish exercise regimes for different IBD patient groups. Additional basic and clinical research with exercise of higher intensities is also needed to establish a potential acceptable limit for physical activity in IBD patients.

## Figures and Tables

**Table 1:** Characteristics of studies assessing the impact of physical activity on onset of IBD included in review.

Reference	Number of subjects	Study characteristics	Study outcome
Sonnenberg, 1990 [28]	12 014	Retrospective study (Germany)	Occupations characterized by outdoor physical work appeared to be protective compared with those occupations classified as sedentary against the onset of IBD

Persson et al., 1993 [29]	152 CD 145 UC 305 controls	Postal questionnaire (Sweden, Stockholm County)	CD onset (but not UC) inversely related to weekly and daily exercise five years before disease onset

Boggild et al., 1996 [30]	2 273 872	A cohort, comprising male and female aged 20–59 (January 1986), was followed up for hospitalizations due to chronic IBD until December 31, 1990 (Denmark)	Sedentary office work may contribute to IBD onset

Klein et al., 1998 [31]	55 UC 33 with CD 76 population and 68 clinic controls	Preillness lifestyle patterns compared among recently diagnosed IBD patients and matched population and clinic controls (Israel)	IBD patients had lower levels of physical activity during their preillness period compared to clinic but not population controls

Cucino and Sonnenberg, 2001 [32]	2399 CD and 2419 UC	Examined the occupations of IBD mortalities 1991–1996 (USA)	IBD mortality was low in occupations associated with manual work and relatively high in sedentary occupations in both CD and UC

Halfvarson et al., 2006 [33]	Discordant twin pairs 102 CD 125 UC	Population-based twin cohort using the cotwin control method through a postal questionnaire (Sweden, Denmark)	No significant difference found in physical activity between twins pairs

Chan et al., 2013 [34]	300 724	Anthropometric measurements of height and weight plus physical activity and total energy intake from validated questionnaires taken at recruitment. The cohort was monitored identifying participants who developed either CD or UC (European Prospective Investigation into Cancer and Nutrition study.).	No association was found between physical activity and onset of IBD

Hlavaty et al., 2013 [35]	190 CD 148 UC 355 controls	Case-control study through questionnaire (Slovakia)	CD and UC associated with less than two sporting weekly activities

Khalili et al., 2013 [36]	194 and 711 women	Women enrolled in this prospective cohort study provided data on physical activity since 1984 in the Nurses' Health Study and 1989 in the Nurses' Health Study II and followed up through 2010 (USA).	Physical activity was inversely associated with risk of Crohn's disease but not of ulcerative colitis

**Table 2:** Characteristics of interventional studies examining the impact of exercise on IBD.

Reference	Sample	Disease type	Intervention	Duration of exercise program	Outcome
Robinson et al., 1998 [122]	117 patients: 60 exercise 57 control	CD	Home based low impact exercises program of increasing intensity focused on the hip and lumbar regions	Twice a week (at least 10 monthly), 12 months	Bone mineral density increased in compliant patients in the lumbar spine and the hip

D'Incà et al., 1999 [42]	6 patients in remission 6 control	CD	Acute exercise at 60% of VO_2max_ (cycle ergometer)	One hour	Exercise did not elicit subjective symptoms or changes in intestinal permeability and lipid peroxidation

Loudon et al., 1999 [38]	12 patients with inactive or mildly active disease	CD	Low-intensity walking program subjects walked an average of 2.9 sessions/wk, at an average of 32.6 min/session, and for an average distance of 3.5 km/session	A thrice weekly, 12 wk walking program	Stress diminished, physical health, general well-being, and quality of life improved without disease exacerbation

Elsenbruch et al., 2005 [139]	30 patients with inactive disease 30 control	UC	60 h training program: stress management program, light exercise	10 weeks; 6 h/week	Improvement in quality of life in patients with UC in remission, while no effects of therapy on clinical or physiological parameters were found

Ng et al., 2007 [39]	16 patients with inactive disease 16 patients control	CD	Low-intensity walking 30 min at 60% of maximum heart rate	3 times per week during 3 months	Improvement in quality of life and reductions in CD symptoms

Ploeger et al., 2012 [44]	15 pediatric patients in remission 15 controls	CD	Moderate intensity continuous exercise (MICE) High intensity intermittent exercise (HIIE)	30 min of cycling at 50% of peak mechanical power (PMP) 6 bouts of 4 × 15-s of cycling at 100% PMP	No significant exacerbation of the disease or inflammatory cytokine responses in both types of exercise

Chan et al., 2014 [45]	918 IBD patients (54% CD and 46% UC)	CD, UC	UK online survey regarding exercise habits	Regular exercise	72% reported that exercise made them feel better, but 80% had to stop exercising temporarily or permanently at some point because of the severity of their symptoms

**Table 3:** Characteristics of animal studies examining effects of exercise on experimental colitis.

Reference	Species	Study characteristics	Study outcome
Cook et al., 2013 [46]	Male C57Bl/6J mice	Mice were randomly assigned to 3 groups: (1) sedentary, (2) moderate intensity forced treadmill exercise (FTR) (8–12 m/min, 40 min, 6 weeks, and 5x/week), or (3) voluntary wheel training (VWR) (30 days access to wheels). Dextran sodium sulfate (DSS) was given at 2% (w/v) in drinking water over 5 days. Mice discontinued exercise 24 h prior to DSS treatment.	Forced treadmill exercise exacerbated the colitis manifestation and mucosal inflammation (rise in diarrhea and gene expression of IL-6, IL-1*β*, and IL-17 in the colon. Also higher mortality was observed in the FTR/DSS group. VWR alleviated colitis symptoms and reduced inflammatory gene expression in the colonic mucosa of DSS-treated mice.

Saxena et al., 2012 [48]	Male adiponectin knockout (APNKO) and wild type (WT) mice (C57BL/6)	APNKO and WT mice were randomly assigned to different groups: (1) sedentary (SED); (2) exercise trained (ET); (3) sedentary with dextran sodium sulfate (DSS) treatment (SED + DSS); and (4) exercise trained with DSS (ET + DSS). Exercise-trained mice ran at 18 m/min for 60 min, 5 d/wk for 4 weeks. Subsequently, the ET + DSS and the SED + DSS mice received 2% DSS in their drinking water for 5 days (d), followed by 5 d of regular water.	The clinical symptoms of colitis were unaffected by exercise and there was no difference between the APNKO and WT mice. The clinical symptoms of the DSS-treated APNKO mice were worse than WT mice treated with DSS and had increased local STAT3 activation, higher IL-6, TNF-*α*, IL-1*β*, and IL-10. Exercise training significantly decreased proinflammatory cytokines including IL-6, TNF-*α*, and IL-1*β* and the phosphorylation expression of STAT3 in both WT and APNKO mice in DSS + EX.

Hoffman-Goetz et al., 2010 [49]	Female C57BL/6 mice	Animals were given 16 weeks of wheel running (WR) or a control condition and at the end of training were assigned to a single acute treadmill exercise session (30 min at 22 m/min, 30 min at 25 m/min, and 30 min at 28 m/min).	WR mice had lower TNF-*α* and caspase 7 and higher IL-10 and IL-6 expression in intestinal lymphocyte (ILymph) than No WR mice. A single exposure to intense aerobic treadmill exercise increased pro-(TNF-*α*) and anti-(IL-10) inflammatory cytokine and proapoptotic protein (caspase 3) expression in ILymph. The percent of early and late apoptotic and dead ILymph were higher after acute exercise.

Luo et al., (2013) [52]	Male Balb/c mice	Effect of moderate exercise (30 min per day swimming) on repeated restraint stress- (RRS-) induced intestinal barrier dysfunction.	Exercise attenuated chronic stress-induced intestinal barrier dysfunction in mice, possibly due to augmentation of antimicrobial responses in the small intestine.
